# A Cross‐Nationally Validated Nomogram for Cardiometabolic Multimorbidity in Overweight/Obese Older Adults: CHARLS and HRS Cohorts

**DOI:** 10.1111/dom.71077

**Published:** 2026-07-06

**Authors:** Yanhan Wei, Siyi He, Huizhen Chen, Yanzhong Wang

**Affiliations:** ^1^ Institute of Health Informatics, University College London London UK; ^2^ School of Life Course and Population Sciences, King's College London London UK; ^3^ Chengdu University of Traditional Chinese Medicine, Clinical Medical School Chengdu China

**Keywords:** cardiometabolic multimorbidity, CHARLS, nomogram, overweight and obesity, predictive model

## Abstract

**Aims:**

Middle‐aged and older adults with overweight or obesity are at increased risk of cardiometabolic multimorbidity (CMM), yet validated prediction tools tailored to this population remain limited. This study aimed to develop and validate a multidimensional nomogram integrating metabolic, functional and psychological predictors.

**Materials and Methods:**

Using the China Health and Retirement Longitudinal Study (CHARLS) data (2011 and 2015), 3965 overweight/obese adults with a body mass index (BMI) ≥ 24.0 kg/m^2^ were randomly split into development (*n* = 2775) and validation (*n* = 1190) sets. Candidate predictors spanned socio‐demographic, lifestyle, functional, psychological and biochemical factors. External validation included 8180 overweight/obese participants from the US Health and Retirement Study (HRS). Least absolute shrinkage and selection operator and multivariable logistic regression were applied to build a nomogram. Performance was assessed by area under the curve (AUC), calibration and decision curve analysis.

**Results:**

CMM prevalence was 3.91%. Eight variables were retained: residence, Activities of daily living (ADL) score, the Center for Epidemiologic Studies Depression Scale (CESD‐10) score, haemoglobin A1c (HbA1c), hypertension, arthritis, dyslipidaemia and memory disorder. AUCs were 0.871 (95% CI: 0.838–0.905) (development), 0.817 (95% CI: 0.768–0.867) (validation) and 0.733 (95% CI: 0.714–0.751) in HRS external validation, with acceptable calibration in CHARLS and evidence of underestimation in HRS that improved after intercept recalibration, and clear net benefit. Positive predictive values were additionally reported to support clinical interpretation under the low CMM prevalence.

**Conclusions:**

This nomogram integrating metabolic, functional and psychological predictors demonstrated strong discrimination in the CHARLS cohort and maintained adequate discriminative performance in a cross‐national external validation using the US HRS cohort despite differences in population characteristics and measurement instruments, supporting its potential utility for early CMM risk stratification in overweight/obese older adults. The external HRS validation showed fair discrimination, suggesting that model recalibration may be necessary before broad cross‐national implementation.

**Abbreviations**
ADL: activities of daily living
AUC: area under the curve
AUROC: area under the receiver operating characteristic curve
BMI: body mass index
CESD‐10: 10‐item Center for Epidemiologic Studies Depression Scale
CHARLS: China Health and Retirement Longitudinal Study
CMM: cardiometabolic multimorbidity
CRP: C‐reactive protein
DBP: diastolic blood pressure
DCA: decision curve analysis
HbA1c: haemoglobin A1c
HDL‐C: high‐density lipoprotein cholesterol
LASSO: least absolute shrinkage and selection operator
LDL‐C: low‐density lipoprotein cholesterol
SBP: systolic blood pressure
TC: total cholesterol
TG: triglycerides
TICS: telephone Interview for Cognitive Status
UA: uric acid
WC: waist circumference

## 1 Introduction

Cardiometabolic diseases, including diabetes, coronary heart disease and stroke, are leading causes of death and disability worldwide, imposing substantial societal and healthcare burdens [1, 2]. In China, rapid population ageing and lifestyle transitions have been accompanied by rising prevalence of diabetes, heart disease and stroke among middle‐aged and older adults, making these conditions a major public health concern [3, 4, 5].

Obesity is a key modifiable risk factor for these disorders. Extensive epidemiologic evidence shows that overweight and obesity markedly increase the risk of diabetes [6, 7] and are important risk factors for heart disease [8, 9] and stroke [10]. In Chinese populations, evidence‐based body mass index (BMI) thresholds indicate elevated metabolic risk at BMI ≥ 24 kg/m^2^ and substantially higher risk at BMI ≥ 28 kg/m^2^ [11, 12]. Consequently, overweight/obese middle‐aged and older adults constitute a priority group for cardiometabolic risk prevention.

The construct of cardiometabolic multimorbidity (CMM), the coexistence of any two or more of diabetes, heart disease and stroke, has gained increasing attention [13]. Compared with single conditions, CMM is associated with substantially higher mortality [13, 14, 15], adverse functional outcomes [16] and greater healthcare utilisation and economic burden [17, 18]. Early identification of individuals at high risk of CMM is therefore essential to enable targeted prevention and efficient resource allocation.

However, most existing CMM prediction studies either target general older populations [19] or focus on patients with a single index disease [20], leaving a gap for tools tailored to overweight/obese middle‐aged and older adults. To address this gap, we used nationally representative data from the China Health and Retirement Longitudinal Study (CHARLS) [21] to develop and validate a pragmatic risk‐prediction model for CMM in this high‐priority group. We integrated socio‐demographic, lifestyle, functional, psychological and biochemical variables; applied least absolute shrinkage and selection operator (LASSO) for feature selection; and constructed a multivariable logistic‐regression nomogram. Model performance was assessed in terms of discrimination, calibration and clinical utility, aiming to provide an early identification tool to inform community and clinical interventions for overweight/obese Chinese adults.

## 2 Materials and Methods

### 2.1 Data Source

Data for this study were obtained from the China Health and Retirement Longitudinal Study (CHARLS, http://charls.pku.edu.cn). CHARLS is an ongoing nationally representative longitudinal survey targeting individuals aged 45 years and older, covering 150 counties/districts and 450 communities/villages across 28 provinces, autonomous regions and municipalities in China. It systematically collects detailed information on demographics, socioeconomic status, health, pension and related domains. The study protocol was approved by the Biomedical Ethics Committee of Peking University (IRB00001052‐11015), and all participants provided written informed consent in accordance with the principles of the Declaration of Helsinki. In the present analysis, individuals who participated for the first time in the 2011 and 2015 survey waves were included as the baseline population. After restricting the sample to overweight or obese individuals with complete CMM outcome data (*n* = 5487) and further excluding those with missing key laboratory measurements, a total of 3965 individuals were retained in the final analytic cohort. The study flowchart is presented in Figure S1.

### 2.2 Definition of Overweight/Obesity

In this study, overweight and obesity were defined according to the BMI, which was calculated as weight (kg) divided by height squared (m^2^). Based on the recommended criteria of the Working Group on Obesity in China (WGOC) [11], overweight was defined as BMI ≥ 24.0 kg/m^2^ and obesity as BMI ≥ 28.0 kg/m^2^. Therefore, individuals with overweight or obesity were classified as those with BMI ≥ 24.0 kg/m^2^. These BMI cut‐offs were supported by Chinese population‐based evidence from the Working Group on Obesity in China and by international guidance indicating that Asian populations experience metabolic risk at lower BMI levels [11, 12].

### 2.3 Assessment of CMM

Consistent with previous studies [13, 22], participants were asked whether they had ever been diagnosed by a doctor with (1) diabetes or high blood sugar, (2) heart disease (including heart attack, angina, coronary heart disease or heart failure) or (3) stroke. CMM in this study was defined as the presence of two or more of these conditions.

For the CHARLS cohort, diabetes was defined by self‐reported physician diagnosis of diabetes or high blood sugar; heart disease included self‐reported physician diagnosis of heart attack, angina, coronary heart disease, heart failure or other heart problems; and stroke was defined by self‐reported physician diagnosis of stroke. A disease count was calculated by summing these three components, and CMM was coded as present when participants had any two or more of the three conditions. This disease‐count definition is consistent with prior CMM research using diabetes, heart disease/coronary heart disease and stroke as the core cardiometabolic conditions [13, 22]. Participants with missing information on any of the three CMM components were excluded before outcome definition.

### 2.4 Socio‐Demographic Information

Socio‐demographic variables included age, sex, marital status, educational level, residence and health insurance status. Sex was categorised as female or male. Marital status was classified as married or other (including unmarried, divorced or widowed). Educational level was categorised into four groups: below primary school, primary school, middle school and high school or above. Residence was classified as urban or rural according to household registration. Insurance was recorded as yes or no.

### 2.5 Health‐Related Information

Within the health‐related data examined as potential risk factors, participants were asked whether they had ever been diagnosed by a doctor with a series of chronic conditions. These included hypertension, dyslipidaemia, liver disease, kidney disease, arthritis, mental illness, memory disorder, headache, chest pain and back pain, each coded as a binary variable (yes/no). In addition, lifestyle factors were assessed. Smoking status was categorised as never, former or current smoker, while drinking status was classified as never, former or current drinker. Health insurance coverage was also documented (yes/no). These variables were derived directly from the CHARLS questionnaire and served as important covariates in subsequent analyses.

### 2.6 Assessment of Depressive Symptoms (CESD‐10)

Depressive symptoms were assessed using the 10‐item short form of the Center for Epidemiologic Studies Depression Scale (CESD‐10). This scale evaluates the respondent's emotional state and behaviours over the past week. Each item is rated according to the frequency of symptoms as follows: ‘Rarely or none of the time (< 1 day)’, ‘Some or a little of the time (1–2 days)”, ‘Occasionally or a moderate amount of the time (3–4 days)’, and ‘Most or all of the time (5–7 days)’, corresponding to scores of 0, 1, 2 and 3, respectively. The total score ranges from 0 to 30, with higher scores indicating more severe depressive symptoms. In the present study, the CESD‐10 score was analysed as a continuous variable.

### 2.7 Assessment of Cognitive Function

Cognitive function was assessed using the Total cognition score in CHARLS, which was derived from three components: the Telephone Interview for Cognitive Status (TICS), Word Recall and Picture Drawing. The TICS evaluated orientation, calculation and attention by asking participants to identify the current year, month, day, day of the week, and season and to perform serial subtractions of 7 from 100, with each correct response scoring 1 point, for a total of 0–10 points. Episodic memory was measured by Word Recall, where participants were required to recall a list of 10 words immediately and again after a short delay, with each correct recall scoring 1 point; the average of the immediate and delayed recall was calculated to yield a score of 0–10 points. Visuospatial ability was assessed through Picture Drawing, in which participants were asked to reproduce a diagram of two overlapping pentagons, scored as 1 for correct and 0 for incorrect. The three components were summed to generate the Total cognition score, ranging from 0 to 21, with higher scores indicating better cognitive function.

### 2.8 Assessment of Activities of Daily Living

Activities of daily living (ADL) were assessed using the ADL scale, which included six basic tasks: dressing, bathing, eating, getting in and out of bed, toileting and continence. For each task, participants reported the degree of difficulty on a four‐point scale: ‘No, I don't have any difficulty’, ‘I have difficulty but can still do it’, ‘Yes, I have difficulty and need help’, and ‘I cannot do it’, corresponding to scores of 1, 2, 3 and 4, respectively. The total ADL score was calculated by summing across the six items, with higher scores indicating greater functional impairment. In this study, the ADL score was analysed as a continuous variable.

### 2.9 Biochemical Indicators

Biochemical indicators were obtained through physical examinations and laboratory tests. These included blood glucose, serum creatinine, total cholesterol (TC), triglycerides (TG), high‐density lipoprotein cholesterol (HDL‐C), low‐density lipoprotein cholesterol (LDL‐C), C‐reactive protein (CRP), haemoglobin A1c (HbA1c) and uric acid (UA). Anthropometric measurements included waist circumference (WC). Blood pressure was measured three times, and the average values of systolic blood pressure (SBP) and diastolic blood pressure (DBP) were recorded for analysis.

### 2.10 External Test Dataset

For external validation, data were obtained from the Health and Retirement Study (HRS), a nationally representative longitudinal survey of middle‐aged and older adults in the United States [23]. Participants who were first enrolled in the 2006 or 2008 survey waves were considered as the baseline population. A total of 10 578 individuals were initially identified. After excluding participants with missing values in variables required for the prediction model and restricting the analysis to individuals with overweight or obesity (BMI ≥ 24.0 kg/m^2^, consistent with the Chinese criteria used in the development dataset), 8180 participants were retained for the final analysis. The project (study 20‐2429) was reviewed by the University of North Carolina at Chapel Hill Office of Human Research Ethics, which determined that it was not human subjects research as defined under federal regulations and did not require institutional review board approval.

To address the concern that BMI thresholds differ across populations, we retained BMI ≥ 24.0 kg/m^2^ in the main HRS analysis to maintain consistency with the development cohort and additionally performed a sensitivity analysis using the conventional US threshold of BMI ≥ 25.0 kg/m^2^ based on the World Health Organisation definition of overweight [12].

The definition of CMM was consistent with that used in the development cohort. Specifically, CMM was defined as the presence of any two or more of the following three physician‐diagnosed conditions: diabetes or high blood sugar, heart disease and stroke or transient ischaemic attack.

Variable definitions in the external dataset were harmonised as closely as possible with those in CHARLS. ADL scores were assessed using six basic functional items, with a total score ranging from 1 to 6, where higher scores indicate worse functional status. Depressive symptoms were measured using an 8‐item version of the CESD, with a total score ranging from 0 to 8, and higher scores indicating more severe depressive symptoms. Residence was categorised as urban or rural. Hypertension, arthritis and memory disorder were defined based on self‐reported physician diagnoses and treated as binary variables (yes/no). Dyslipidaemia was defined according to established clinical criteria as TC > 200 mg/dL and/or HDL‐C < 40 mg/dL in men or < 50 mg/dL in women. Glycaemic status was assessed using HbA1c levels as a continuous variable.

A detailed harmonisation table comparing CHARLS and HRS definitions for the outcome and final predictors has been added to the Table S3.

### 2.11 Missing Data Handling

Missing data were assessed before model development. After excluding participants with missing CMM outcome data and restricting the analysis to overweight/obese individuals (BMI ≥ 24 kg/m^2^), 5487 participants remained eligible. Because the laboratory biomarkers serve as objective core predictors for which imputation under high missingness would be unreliable, participants with missing values in any key biochemical measurement (including HbA1c, blood glucose and lipid profile) were excluded, yielding a final analytic sample of 3965 participants with complete laboratory data. Within this analytic sample, the remaining missing values in the non‐laboratory variables (including age, systolic and diastolic blood pressure, waist circumference, ADL score, CESD‐10 score and total cognition score) were handled using multiple imputation; variable‐specific missingness before imputation is summarised in Table S10. Missing values in categorical disease‐history and lifestyle variables were coded as absence of the condition when this was consistent with the questionnaire structure and coding framework of CHARLS. The participant selection process and exclusions are shown in Figure S1. For the HRS external validation cohort, complete‐case analysis was performed after harmonisation of all variables required by the final model.

### 2.12 Statistical Analysis

Data from the 2011 and 2015 waves of the CHARLS were used for analysis. Baseline characteristics of the study population were first described. Continuous variables were presented as medians with interquartile ranges [M (Q1, Q3)], and between‐group comparisons were performed using the rank‐sum test. Categorical variables were expressed as frequencies and percentages, with between‐group comparisons conducted using the *χ* ^2^ test or Fisher's exact test, as appropriate.

With CMM as the dependent variable, univariate logistic regression analyses were initially conducted to identify potential associated factors. Candidate variables were then entered into the LASSO regression model to reduce multicollinearity and further select variables. Finally, predictors identified through LASSO were incorporated into multivariate logistic regression analyses to determine independent risk factors for CMM. A nomogram was subsequently developed using the ‘rms’ package in R to visually depict the risk of CMM among overweight or obese older adults in China. For model evaluation, the discriminative ability was assessed using the area under the receiver operating characteristic curve (AUROC). Calibration was evaluated with calibration plots and the Hosmer–Lemeshow goodness‐of‐fit test to examine the agreement between predicted probabilities and observed outcomes. Clinical utility was further assessed using decision curve analysis (DCA). Continuous predictors, including ADL score, CESD score, HbA1c and other biochemical markers, were retained as continuous variables in the modelling process rather than being arbitrarily categorised. Variance inflation factors were calculated for the final predictors to assess multicollinearity. To evaluate parsimony, we also fitted a pared‐down model excluding potentially overlapping frailty‐related predictors. Predictive values at practical risk thresholds were calculated to facilitate clinical interpretation. Two sensitivity analyses were conducted to assess the robustness of the prediction model. First, because depressive symptoms were measured using different scales in the development/internal validation cohorts (CESD‐10, range 0–30) and the HRS external validation cohort (CESD‐8, range 0–8), depressive symptoms were recoded as a binary variable using established cut‐offs (CESD‐10 ≥ 10 in CHARLS [24] and CESD‐8 ≥ 4 in HRS [25]). The prediction model was then refitted and its discrimination was reassessed in the development, validation and external validation datasets. Second, because dyslipidaemia in CHARLS was primarily based on self‐reported physician diagnosis, we evaluated its agreement with a laboratory‐defined dyslipidaemia variable. Laboratory‐defined dyslipidaemia was defined as total cholesterol > 200 mg/dL and/or HDL‐C < 40 mg/dL in men or < 50 mg/dL in women. Agreement between self‐reported and laboratory‐defined dyslipidaemia was assessed using Cohen's kappa coefficient, sensitivity, specificity, positive predictive value and negative predictive value. The study reporting was guided by the TRIPOD statement [26], and a completed TRIPOD checklist is provided as an additional Supporting Information.

All statistical analyses were conducted using R software (version 4.3.3). All tests were two‐sided, and a *p* < 0.05 was considered statistically significant.

## 3 Results

### 3.1 Participant Characteristics

A total of 3965 overweight or obese middle‐aged and older individuals were included in this study. The median age of the overall population was 57 years (interquartile range, 50–63 years), and 1508 participants (38.0%) were male while 2457 (62.0%) were female. Participants were randomly assigned to a development set (*n* = 2775) and a validation set (*n* = 1190). The demographic, lifestyle and clinical characteristics of the two sets are summarised in Table S1. No statistically significant differences were observed between the development and validation sets across the baseline characteristics (all *p* > 0.05).

### 3.2 Prevalence, LASSO‐Based Variable Selection and Independent Risk Factors of CMM

The prevalence of CMM was 3.91% (155/3965) in the total population. In the development dataset, univariate logistic regression analysis identified several variables significantly associated with CMM (Table S2), including Age (*p* < 0.001), Residence (*p* < 0.001), SBP (*p* = 0.003), ADL score (*p* < 0.001), CESD‐10 (*p* < 0.001), Glucose (*p* < 0.001), Creatinine (p < 0.001), TG (*p* = 0.027), HDL‐C (*p* = 0.005), HbA1c (*p* < 0.001), Hypertension (*p* < 0.001), Arthritis (*p* < 0.001), Dyslipidaemia (p < 0.001), Liver disease (*p* = 0.035), Kidney disease (*p* < 0.001), Memory disorder (*p* < 0.001) and Chest pain (*p* < 0.001). These 17 variables were subsequently entered into a LASSO regression model, and the optimal penalty parameter *λ* was determined via tenfold cross‐validation (Figure S2A,B). Eleven candidate predictors were retained and included in the multivariate logistic regression analysis. As shown in Table 1, rural residence compared with urban residence (odds ratio [OR] = 0.353, 95% confidence interval [CI]: 0.223–0.558, *p* < 0.001), ADL score (OR = 1.206, 95% CI: 1.002–1.452, *p* = 0.048), CESD‐10 score (OR = 1.050, 95% CI: 1.014–1.087, *p* = 0.006), HbA1c (OR = 1.565, 95% CI: 1.231–1.991, *p* < 0.001), hypertension (OR = 4.400, 95% CI: 2.638–7.339, *p* < 0.001), arthritis (OR = 1.829, 95% CI: 1.172–2.854, *p* = 0.008), dyslipidaemia (OR = 2.576, 95% CI: 1.636–4.055, *p* < 0.001) and memory disorder (OR = 2.735, 95% CI: 1.039–7.196, *p* = 0.042) were identified as independent risk factors for CMM, while glucose (*p* = 0.225), kidney disease (*p* = 0.075) and chest pain (*p* = 0.198) were not statistically significant in the multivariate analysis. These eight independent predictors were subsequently incorporated into the final nomogram model to quantitatively estimate the probability of CMM among overweight and obese middle‐aged and older adults.

**TABLE 1:** Multivariate Logistic regression results of predictors for CMM in the development dataset.

Variable	OR (95% CI)	Wald *χ* ^2^	*p*
Residence			
Urban	Ref (1.00)		
Rural	0.353 (0.223–0.558)	19.896	< 0.001
ADL score	1.206 (1.002–1.452)	3.914	0.048
CESD‐10	1.050 (1.014–1.087)	7.657	0.006
Glucose	1.003 (0.998–1.009)	1.470	0.225
HbA1c	1.565 (1.231–1.991)	13.326	< 0.001
Hypertension			
No	Ref (1.00)		
Yes	4.400 (2.638–7.339)	32.224	< 0.001
Arthritis			
No	Ref (1.00)		
Yes	1.829 (1.172–2.854)	7.079	0.008
Dyslipidaemia			
No	Ref (1.00)		
Yes	2.576 (1.636–4.055)	16.707	< 0.001
Kidney disease			
No	Ref (1.00)		
Yes	1.819 (0.942–3.512)	3.180	0.075
Memory disorder			
No	Ref (1.00)		
Yes	2.735 (1.039–7.196)	4.155	0.042
Chest pain			
No	Ref (1.00)		
Yes	1.568 (0.79–3.113)	1.654	0.198

Abbreviations: ADL, activities of daily living; CESD‐10, 10‐item Center for Epidemiologic Studies Depression Scale; CI, confidence interval; HbA1c, haemoglobin A1c; OR, odds ratio.

### 3.3 Predictive Model Development

**FIGURE 1:**
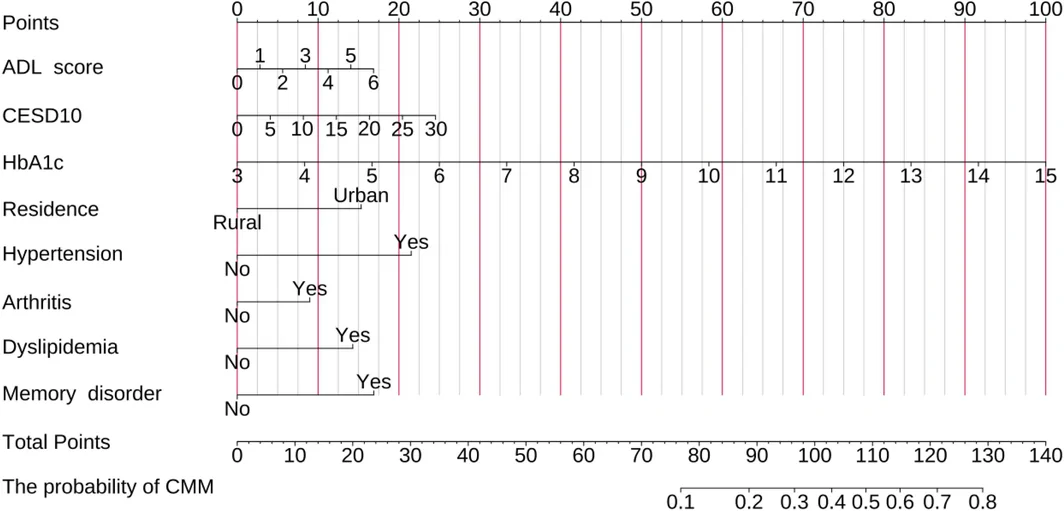
Nomogram for predicting the probability of CMM in overweight/obese middle‐aged and older individuals.

The final predictors included ADL score, CESD‐10 depressive symptoms score, HbA1c, residence, hypertension, arthritis, dyslipidaemia and memory disorder (Figure 1). The predictive model was presented as a nomogram, which can be used to quantitatively estimate the probability of CMM among overweight or obese middle‐aged and older individuals. A web‐based nomogram calculator has also been made available: https://1depression.shinyapps.io/DNomCMM/.

### 3.4 Validation of the Prediction Model

**FIGURE 2:**
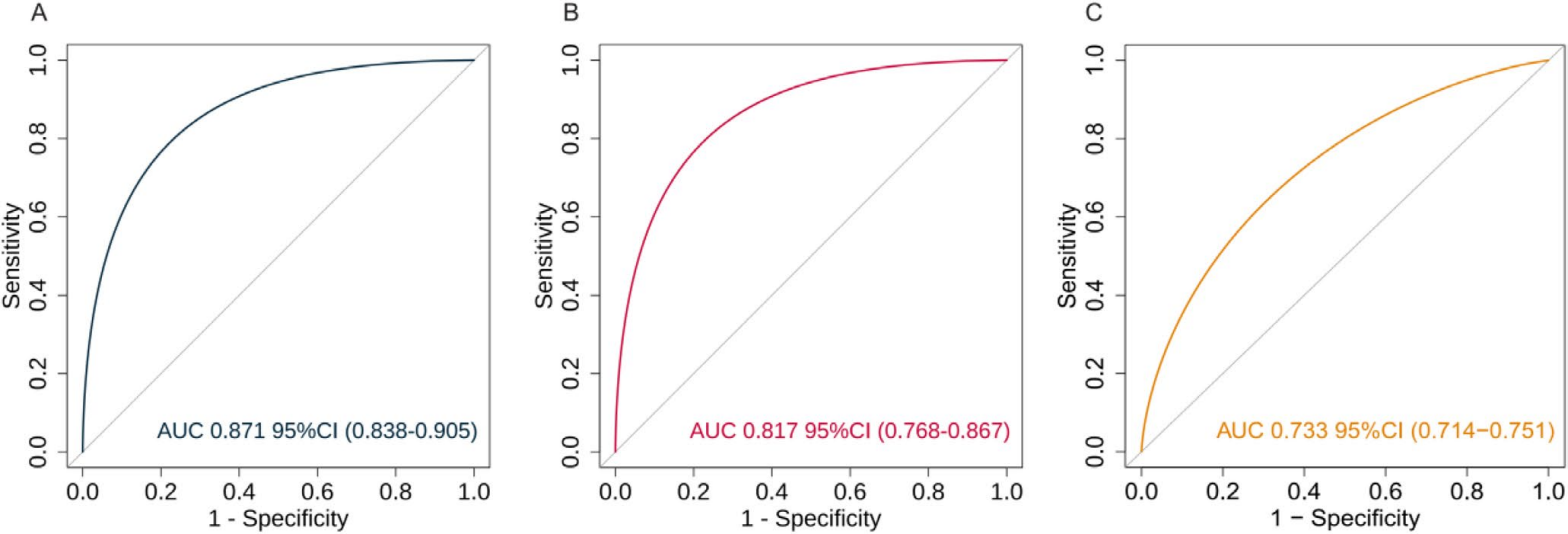
ROC curves for CMM prediction model in overweight/obese middle‐aged and older individuals. (A) ROC curves generated using the development dataset. (B) ROC curves generated using the validation dataset. (C) ROC curves generated using the external test dataset.

To evaluate the ability of the predictive model to distinguish the probability of CMM among overweight/obese middle‐aged and older individuals, the AUC was calculated. As shown in Figure 2, in the development dataset, the predictive model yielded an AUC of 0.871 (95% CI = 0.838–0.905), with a specificity of 0.760 and a sensitivity of 0.874. In the validation dataset, the AUC was 0.817 (95% CI = 0.768–0.867), with a specificity of 0.714 and a sensitivity of 0.808. In the external test dataset, the AUC was 0.733 (95% CI = 0.714–0.751), with a specificity of 0.601 and a sensitivity of 0.768. These findings indicate that the nomogram model possesses good discriminatory power and acceptable predictive value, and can reasonably identify the probability of CMM among overweight/obese middle‐aged and older adults. Multicollinearity among the eight final predictors was low, with all variance inflation factors ranging from 1.02 to 1.15 (Table S5). A pared‐down model excluding ADL score, CESD‐10 score and memory disorder yielded AUCs of 0.850, 0.812 and 0.724 in the development, validation and HRS external validation datasets, respectively, compared with 0.871, 0.817 and 0.733 for the full model (Table S6), supporting retention of the multidimensional model. In the HRS sensitivity analysis using BMI ≥ 25.0 kg/m^2^, 7556 participants were included and the model achieved an AUC of 0.731, with sensitivity of 0.742 and specificity of 0.594 at the Youden‐index threshold (Table S8), supporting the robustness of the external validation results to the BMI definition.

### 3.5 Calibration of the Prediction Model

**FIGURE 3:**
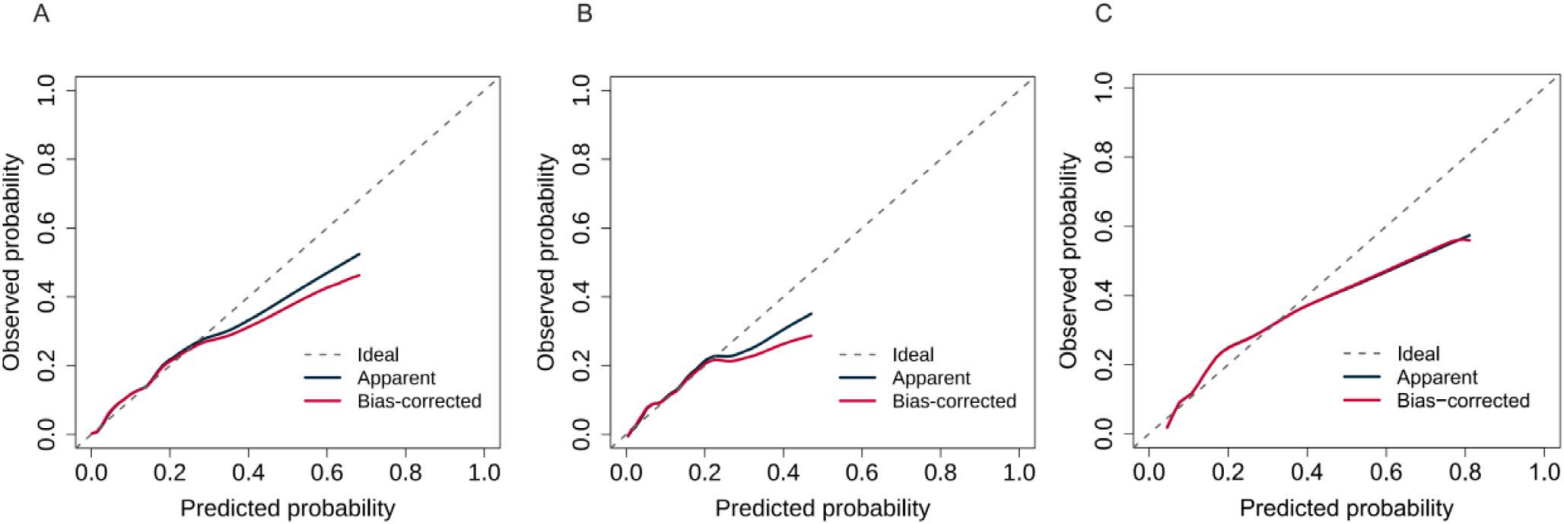
Calibration curves of the CMM prediction model in overweight/obese middle‐aged and older individuals. (A) Calibration curves generated using the development dataset. (B) Calibration curves generated using the validation dataset. (C) Calibration curves generated using the external test dataset.

The goodness of fit of the nomogram was assessed using calibration plots with 1000 bootstrap resamples together with the Hosmer–Lemeshow test. The Hosmer–Lemeshow test was non‐significant in the training (*χ* ^2^ = 8.866, df = 8, *p* = 0.354), validation (*χ* ^2^ = 15.234, df = 8, *p* = 0.055) and external test (*χ* ^2^ = 6.632, df = 8, *p* = 0.577) datasets; however, as this test has limited power to detect systematic miscalibration (calibration‐in‐the‐large), it was interpreted alongside the calibration metrics below rather than as standalone evidence of good external calibration. As shown in Figure 3, calibration was acceptable in the CHARLS development and internal validation datasets. In the HRS external validation cohort, however, additional calibration statistics indicated underestimation of absolute CMM risk (O:E ratio = 1.514, calibration intercept = 0.539, calibration slope = 0.670); after intercept recalibration, the O:E ratio improved to 1.000 (Table S9). These findings suggest that recalibration or local updating may be required before applying the nomogram in non‐Chinese populations.

### 3.6 Assessment of Clinical Effectiveness

**FIGURE 4:**
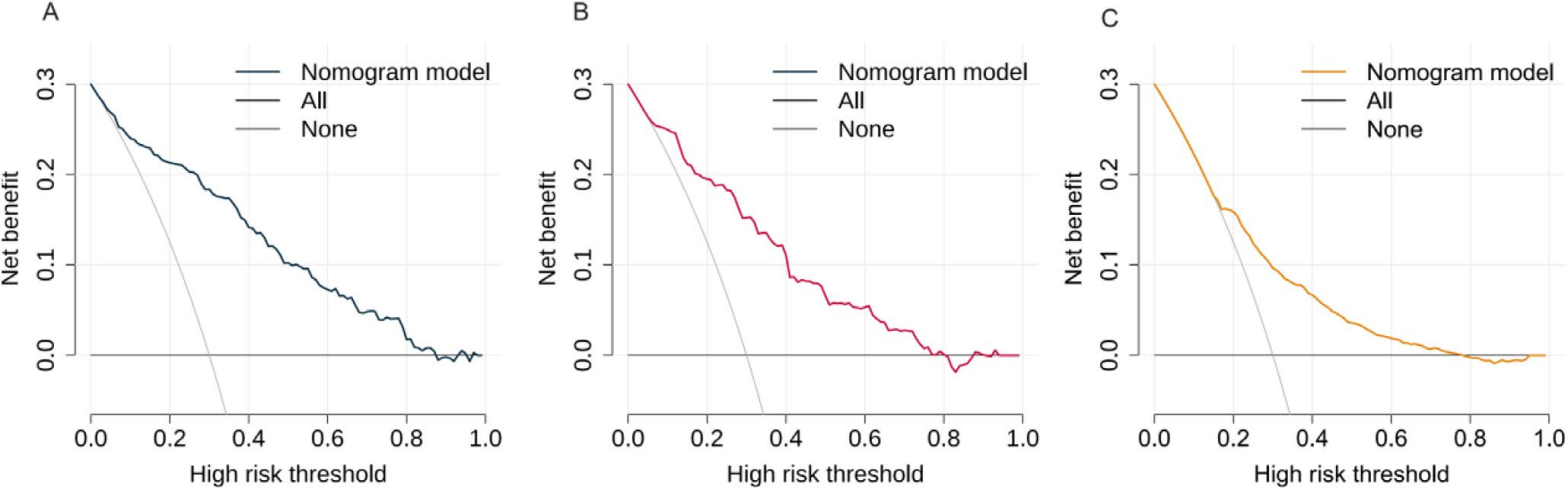
Decision curve plots of the CMM prediction model in overweight/obese middle‐aged and older individuals. (A) DCA curves generated using the development dataset. (B) DCA curves generated using the validation dataset. (C) DCA curves generated using the external test dataset.

The clinical utility of the model was evaluated using DCA, as illustrated in Figure 4. The decision curves demonstrate that the nomogram model provides a greater net benefit across a range of threshold probabilities compared with the ‘treat‐all’ or ‘treat‐none’ strategies, indicating favourable predictive performance and clinical applicability. Because the prevalence of CMM was low, we additionally reported predictive values at practical thresholds (Table S7). In the development set, PPV increased from 12.3% at the Youden‐index threshold to 30.9% at a 20% predicted‐risk threshold, while NPV remained high (97.3%–99.4%). Similar patterns were observed in the validation set. In the HRS external validation dataset, PPV increased from 17.0% to 22.5% across the same thresholds, while NPV remained between 93.6% and 96.7%. These findings indicate that the model may be more useful for ruling out low‐risk individuals and prioritising higher‐risk individuals for further assessment than for making a definitive diagnosis.

### 3.7 Sensitivity Analysis

Sensitivity analyses supported the robustness of the prediction model. After recoding depressive symptoms as a binary variable using cohort‐specific CESD cut‐offs, model discrimination remained largely unchanged across the development, validation and HRS external validation datasets compared with the primary analysis (Figure S3), indicating that differences in CESD scale structure had minimal impact on predictive performance. Agreement between self‐reported and laboratory‐defined dyslipidaemia was further evaluated in CHARLS (Table S4). Laboratory‐defined dyslipidaemia was substantially more prevalent than self‐reported dyslipidaemia (76.6% vs. 15.8%). Agreement between the two definitions was poor (*κ* = 0.034), with sensitivity and specificity of 17.3% and 89.1%, respectively. These findings suggest that self‐reported dyslipidaemia may underestimate the true burden of lipid abnormalities and could contribute to some degree of predictor misclassification. Nevertheless, the overall model performance remained stable, supporting the robustness of the nomogram despite differences in variable definitions between cohorts.

## 4 Discussion

Using nationally representative CHARLS data, this study is the first to develop and validate a risk‐prediction model for CMM among overweight and obese middle‐aged and older adults in China. The final model incorporated eight independent predictors (hypertension, dyslipidaemia, HbA1c, ADL score, CESD‐10 score, memory disorder, residence and arthritis) and demonstrated strong discrimination in the development and validation sets (AUCs of 0.871 and 0.817) and only fair discrimination on external validation (AUC 0.733), acceptable calibration in the CHARLS cohorts, and clear net clinical benefit on decision‐curve analysis. These findings suggest that the nomogram can serve as a practical tool for risk stratification of individuals at high risk of CMM in clinical and community settings. In light of the HRS AUC of 0.733, we now interpret the external performance as fair rather than strong.

In our population, the prevalence of CMM was 3.91%, notably higher than the 2.03% reported by Han et al. in the Beijing Health Management Cohort (BHMC) of the general adult population [27], underscoring the heightened vulnerability of older adults with obesity. The China Kadoorie Biobank (CKB) likewise indicates that CMM represents a common chronic disease pattern in China and is associated with the highest mortality risk [28]. Evidence from Europe aligns with these observations: in the Whitehall II cohort, midlife lifestyle and socioeconomic factors substantially influence cumulative CMM risk [29], while multicohort analyses across Europe and Australia show that obesity, smoking and insufficient physical activity markedly increase CMM risk [30]. US data similarly demonstrate a dose–response relationship between increasing BMI and CMM [30]. Together, these findings reinforce the necessity of risk prediction specifically in older adults with overweight/obesity.

Regarding predictor interpretation, traditional metabolic risk factors remain core drivers of CMM. Hypertension and Dyslipidaemia significantly increased CMM risk, with plausible mechanisms involving endothelial dysfunction, inflammation and accelerated atherosclerosis [31, 32, 33]. Elevated HbA1c reflects long‐term glycaemic dysregulation and is closely linked to diabetes and cardiovascular risk; studies from the UK Biobank and from China have associated HbA1c with both CMM and mortality [34, 35]. Beyond metabolic factors, functional and mental health indicators were also important. Higher ADL score indicates functional limitation/frailty and is strongly associated with clustering of chronic diseases [36]; depressive symptoms (CESD‐10 score) may elevate risk via inflammatory pathways and adverse health behaviours, consistent with systematic reviews and meta‐analyses linking depression to multimorbidity [37, 38]. Memory disorder suggests shared mechanisms between cognitive decline and CMM—such as chronic inflammation, cerebrovascular injury and insulin resistance [39, 40]. In addition, socio‐demographic and inflammatory disease‐related factors were relevant. Residence differences showed lower CMM risk in rural than urban settings, potentially reflecting disparities in healthcare access and under‐diagnosis [41, 42]. Arthritis, as a chronic inflammatory disease, may amplify metabolic risk through systemic inflammation and activity limitation, aligning with the notion that chronic low‐grade inflammation promotes multimorbidity [43]. Collectively, these results indicate that CMM is intertwined not only with metabolic risk factors but also with functional status, psychological health and inflammatory states, highlighting the need for multidimensional risk assessment.

Nevertheless, residence effects should be interpreted cautiously because CMM and several comorbidities were self‐reported. Differential healthcare access and diagnostic awareness between urban and rural participants may have influenced disease ascertainment, potentially amplifying residence‐related associations and attenuating associations for some biomarkers.

Compared with prior work, our study differs in both target population and predictor set. The BHMC model by Han et al. focused on the general adult population with predictors centred on lifestyle and routine clinical indices, whereas our model targets older adults with overweight/obesity and, for the first time, incorporates ADL score, depressive symptoms and memory disorder, which better reflect geriatric contexts [27]. International studies (e.g., Whitehall II and European cohorts reported in BMC Medicine) [29, 30] primarily emphasised lifestyle factors and less often included geriatric functional or psychological features. Our predictor design is therefore more tailored and innovative for ageing populations with excess adiposity, expanding the scope of CMM risk prediction.

The external validation results should also be viewed in the context of cross‐national differences in healthcare systems, lifestyle, socioeconomic conditions and measurement instruments. The HRS AUC of 0.733 indicates fair discrimination, suggesting that recalibration or local updating may be needed before applying the nomogram in non‐Chinese populations.

These cross‐national differences merit closer consideration. China and the United States differ substantially in the organisation of and access to health care: in China, a tiered, household‐registration‐based system and persistent urban–rural disparities in the availability of diagnostic services [41, 42] may lead to differential detection of cardiometabolic conditions, which could partly explain the strong residence effect observed in CHARLS and the attenuation of some biomarker associations. The US HRS population, by contrast, is embedded in an insurance‐based system with different screening intensity and case ascertainment, which—together with an older age structure and the inclusion of transient ischaemic attack in the stroke definition—may alter both the apparent prevalence of CMM and the relative weight of individual predictors. Dietary patterns and body‐composition differences are also relevant: Asian populations tend to develop cardiometabolic risk at lower BMI levels than Western populations [11, 12], which underpins the lower BMI threshold adopted here and implies that the magnitude of obesity‐related associations may not transport directly across settings. Differences in the socioeconomic composition of older adults, education, income, retirement support and health literacy, further shape both the burden of multimorbidity [29] and the behaviour of risk factors. Collectively, these structural, dietary and socioeconomic contrasts offer a plausible explanation for the decline to only fair external discrimination and reinforce that the nomogram should be recalibrated or locally updated, and interpreted within the specific health‐system context, before application outside China.

To illustrate practical use, we propose a simple risk‐stratification workflow for community and primary‐care settings. Overweight or obese adults aged 45 years and older would first have their individual CMM probability estimated with the nomogram or the accompanying web calculator (https://1depression.shinyapps.io/DNomCMM/), which requires only routinely available information (residence, ADL and CESD‐10 scores, HbA1c and self‐reported hypertension, dyslipidaemia, arthritis and memory disorder). Because the model retained high negative predictive value (approximately 97%–99%), individuals classified as low risk can be reassured and managed with routine lifestyle advice, avoiding unnecessary investigations. Given the low prevalence of CMM (3.9%), positive predictive value is modest but increases with the chosen threshold, rising from 12.3% at the Youden‐index cut‐off to 30.9% at a 20% predicted‐risk threshold; individuals exceeding a higher threshold (e.g., a predicted probability ≥ 20%) would therefore be prioritised for confirmatory assessment (measurement of blood pressure, blood glucose or HbA1c and lipids) and, where indicated, referral for specialist evaluation and intensified preventive interventions (weight management, glycaemic and blood‐pressure control, and attention to depressive symptoms and functional decline). The nomogram thus functions as a triage and risk‐stratification aid that directs limited resources to those most likely to benefit, rather than as a stand‐alone diagnostic test.

This study has several strengths. It addresses an under‐served but high‐priority group, overweight and obese middle‐aged and older adults, using nationally representative CHARLS data and a deliberately multidimensional predictor set that integrates metabolic, functional (ADL), psychological (depressive symptoms) and cognitive domains alongside routine clinical factors, better reflecting the geriatric context than models based on demographic and laboratory variables alone. The analytic approach was rigorous and transparent, combining LASSO with multivariable logistic regression to limit overfitting and multicollinearity [44], a parsimony check and a comprehensive evaluation of discrimination, calibration (including calibration slope, intercept and recalibration) and clinical utility by decision‐curve analysis. The model was further validated and recalibrated in an independent US cohort (HRS) and is provided as a practical nomogram with an accompanying web calculator to facilitate clinical and community use.

Several limitations warrant consideration. First, CMM and its component comorbidities were self‐reported, introducing recall bias and probable under‐ascertainment, and diagnostic criteria differed across survey waves and between countries. Second, complete‐case external validation may have introduced selection bias. Third, the cross‐sectional design means the nomogram performs concurrent risk stratification rather than prospective prediction, and several predictors (HbA1c, hypertension and dyslipidaemia) overlap conceptually with components of the CMM outcome, which may have inflated apparent discrimination. Fourth, family history, medication use, physical activity and diet were not included because they were incompletely recorded or not comparably defined across CHARLS and HRS; a parsimonious model using routinely available variables was therefore prioritised, and these established correlates of CMM [30] should be evaluated in future harmonised data. Fifth, some predictor definitions differed across cohorts: depressive symptoms were measured with an 8‐item CESD scale in HRS, and dyslipidaemia was self‐reported in CHARLS but laboratory‐defined in HRS, with poor agreement between the two definitions (*κ* = 0.034) indicating that self‐report substantially underestimated lipid abnormalities; this misclassification may have contributed to the reduced discrimination observed in the HRS external validation. Sixth, with only 103 events and a single random split without bootstrap optimism correction, internal performance may be modestly optimistic, although LASSO penalisation mitigates overfitting. Future multicentre, longitudinal validation and integration of multi‐omics data are warranted.

## 5 Conclusion

In this study, we established a nomogram model based on the CHARLS database to predict the risk of cardiometabolic multimorbidity in overweight and obese middle‐aged and older adults. The model demonstrated good discrimination with ROC analysis (AUC = 0.871 in the development dataset and 0.817 in the validation dataset), satisfactory calibration and clear net clinical benefit from DCA. These results indicate that the model holds potential clinical value for risk stratification of individuals at high risk. The model should be used as a risk‐stratification aid rather than a stand‐alone diagnostic tool, especially in settings outside China where recalibration may be required.

## Author Contributions

Y.H.W. drafted the manuscript and revised it critically. S.H. performed the statistical analyses. H.C. extracted the data, revised the analysis code and contributed to manuscript revision. Y.Z.W. supervised the study and critically revised the manuscript for important intellectual content. All authors read and approved the final manuscript.

## Funding

This research was supported by the National Science and Technology Major Project (2024ZD0523300).

## Ethics Statement

This retrospective study was conducted using de‐identified data from the CHARLS database. All personal identifiers had been removed prior to analysis; therefore, no additional informed consent from participants was required and no ethical concerns were raised. The original CHARLS protocol was approved by the Biomedical Ethics Review Committee of Peking University (IRB00001052–11015), and all participants provided written informed consent at enrolment. The present study was performed in accordance with the principles of the Declaration of Helsinki. For the external‐validation cohort, the analysis of de‐identified Health and Retirement Study data (study 20‐2429) was reviewed by the University of North Carolina at Chapel Hill Office of Human Research Ethics, which determined that it was not human subjects research and did not require institutional review board approval.

## Conflicts of Interest

The authors declare no conflicts of interest.

## Supporting information


**Table S1:** Baseline characteristics of the development dataset and validation dataset.
**Table S2:** Univariate Logistic regression results of predictors for CMM in the development dataset.
**Table S3:** Harmonisation of outcome and predictor definitions between CHARLS and HRS.
**Table S4:** Agreement between self‐reported and laboratory‐defined dyslipidaemia in the CHARLS cohort.
**Table S5:** Variance inflation factors for predictors included in the final nomogram model.
**Table S6:** Discrimination of the full nomogram model and pared‐down model.
**Table S7:** Predictive values at practical thresholds in the development, validation and external validation datasets.
**Table S8:** HRS external validation sensitivity analysis using BMI ≥ 25 kg/m^2^.
**Table S9:** Model discrimination and calibration performance in the development, internal validation and HRS external validation datasets.
**Table S10:** Variable‐specific missing proportions before imputation among overweight/obese CHARLS participants (*n* = 3965). Laboratory biomarkers are complete (0%) by design, as participants with missing laboratory measurements were excluded during sample selection; the remaining continuous non‐laboratory variables were handled by multiple imputation, whereas missing categorical disease‐history and lifestyle responses were coded as absence of the condition.
**Figure S1:** The study's design and flow chart.
**Figure S2:** LASSO regression analysis for analytical screening of predictor variables.
**Figure S3:** ROC curves of the sensitivity analysis using dichotomized depressive symptoms for predicting CMM in overweight/obese middle‐aged and older adults. (A) ROC curve generated using the development dataset. (B) ROC curve generated using the validation dataset. (C) ROC curve generated using the HRS external validation dataset.

**Data S1:** Completed TRIPOD checklist.

## Data Availability

The datasets generated and analysed for the current study are available in the CHARLS repository at http://charls.pku.edu.cn and HRS website (https://hrs.isr.umich.edu/data‐products/).
