# Supplementary material for: A Cross‐Nationally Validated Nomogram for Cardiometabolic Multimorbidity in Overweight/Obese Older Adults: CHARLS and HRS Cohorts
Source: Diabetes Obes Metab. 2026 Jul 6;28(10):8825–35. doi: 10.1111/dom.71077 (PMC13538773; doi:10.1111/dom.71077)
Supplement: Supplementary file 1 [file DOM-28-8825-s001.pdf]

Completed TRIPOD Checklist

Manuscript: A Cross-Nationally Validated Nomogram for Cardiometabolic Multimorbidity in Overweight/Obese Older Adults: CHARLS and HRS Cohorts

The checklist was completed according to the revised manuscript and supplementary materials. (Updated to reflect the analyses added in this revision: multiple imputation for missing data, external calibration with calibration slope, intercept and observed-to-expected ratio, intercept recalibration, and sensitivity analyses.)

Section/Topic	Item	Checklist item	Reported location
Title/Abstract	1	Identify the study as developing and/or validating a multivariable prediction model, the target population, and the outcome to be predicted.	Title; Abstract
Abstract	2a	Provide summary of objectives, study design, setting, participants, sample size, predictors, outcome, statistical analysis, and results.	Abstract
Abstract	2b	Provide performance measures for the model.	Abstract; Results
Introduction	3a	Explain medical context, rationale, and clinical need for the prediction model.	Introduction
Introduction	3b	Specify the study objectives, including model development, internal validation, and external validation.	End of Introduction
Methods	4a	Describe the source of data and study design.	Materials and Methods 2.1; 2.10
Methods	4b	Specify key study dates or survey waves.	Materials and Methods 2.1; 2.10
Methods	5a	Specify key elements of study setting.	Materials and Methods 2.1; 2.10
Methods	5b	Describe eligibility criteria and participant selection.	Materials and Methods 2.1–2.2; Supplementary Figure 1
Methods	6a	Clearly define the outcome predicted by the model, including how and when it was assessed.	Materials and Methods 2.3; 2.10; Supplementary Table 3

Section/Topic	Item	Checklist item	Reported location
Methods	6b	Report any actions to blind assessment of the outcome to predictors if applicable.	Not applicable; secondary analysis of survey data
Methods	7a	Clearly define all predictors used in model development and validation.	Materials and Methods 2.4–2.10; Supplementary Table 3
Methods	7b	Report any actions to blind assessment of predictors to outcome if applicable.	Not applicable; secondary analysis of survey data
Methods	8	Explain sample size and number of outcome events.	Results 3.1–3.2; Supplementary Figure 1
Methods	9	Describe how missing data were handled.	Materials and Methods 2.11 (key laboratory predictors handled by complete-case exclusion; multiple imputation for residual missingness in non-laboratory variables; complete-case for HRS); Supplementary Table 10; Supplementary Figure 1
Methods	10a	Describe model development method, including predictor selection.	Materials and Methods 2.12; LASSO and multivariable logistic regression
Methods	10b	Describe internal validation approach.	Random development/validation split; ROC, calibration, DCA
Methods	10c	For external validation, describe how predictions were calculated.	Materials and Methods 2.10 (External test dataset); 2.12
Methods	10d	Specify performance measures.	AUC, sensitivity, specificity; calibration plot, calibration slope and intercept, observed-to-expected (O:E) ratio, and Hosmer–Lemeshow test; DCA; PPV and NPV
Methods	10e	Describe model updating or recalibration if performed.	**Intercept recalibration performed in the external (HRS) cohort; Results 3.5; Supplementary Table**

Section/Topic	Item	Checklist item	Reported location
			**9**
Methods	11	Provide risk groups if created and explain their choice.	No fixed risk groups created; practical thresholds reported for interpretation
Methods	12	Describe all statistical analyses.	Materials and Methods 2.12, including two sensitivity analyses (CESD recoding; self-reported vs laboratory-defined dyslipidemia)
Results	13a	Describe participant flow through the study.	Results 3.1; Supplementary Figure 1
Results	13b	Describe characteristics of participants and predictors.	Results 3.1; Supplementary Table 1
Results	13c	Report outcome frequency.	Results 3.2
Results	13d	Report unadjusted associations if relevant.	Supplementary Table 2
Results	14a	Specify number of participants and outcome events included in each analysis.	Results 3.1–3.2; External validation section
Results	14b	Report model development results, including selected predictors.	Results 3.2–3.3; Table 1
Results	15a	Present the full prediction model.	Figure 1; Table 1; web calculator link
Results	15b	Explain how to use the prediction model.	Figure 1; web calculator link
Results	16	Report model performance in development, validation, and external validation datasets.	Results 3.4–3.7; Figures 2–4; Supplementary Tables 5–9
Discussion	17	Discuss study limitations, including data source, missing data, self-report, and transportability.	Discussion (limitations paragraph)
Discussion	18	Interpret results in the context of existing evidence.	Discussion
Discussion	19	Discuss implications for practice and future research.	Discussion; Conclusion
Other	20	Give supplementary information and	Supplementary materials; web

Section/Topic	Item	Checklist item	Reported location
		resources.	calculator
Other	21	State where study protocol or data can be accessed if available.	Data availability statement
Other	22	Provide funding source and role of funders.	Funding statement
