# Supplementary material for: A Cross‐Nationally Validated Nomogram for Cardiometabolic Multimorbidity in Overweight/Obese Older Adults: CHARLS and HRS Cohorts
Source: Diabetes Obes Metab. 2026 Jul 6;28(10):8825–35. doi: 10.1111/dom.71077 (PMC13538773; doi:10.1111/dom.71077)
Supplement: Supplementary file 2 [file DOM-28-8825-s002.docx]

**Supplementary Table 1. Baseline characteristics of the development dataset and validation dataset.**

| Variable | All  （N=3,965） | Development set  (N=2,775) | Validation set  (N=1,190) | Z/X^2^ | P value |
| --- | --- | --- | --- | --- | --- |
| Age, years | 57.00 [50.00;63.00] | 57.00 [50.00;63.00] | 57.00 [49.25;63.00] | 0.279 | 0.780 |
| Sex, N(%) |  |  |  | 0.004 | 0.948 |
| Female | 2457 (61.97%) | 1721 (62.02%) | 736 (61.85%) |  |  |
| Male | 1508 (38.03%) | 1054 (37.98%) | 454 (38.15%) |  |  |
| Marital status, N(%) |  |  |  | 0.000 | 1.000 |
| Other | 356 (8.98%) | 249 (8.97%) | 107 (8.99%) |  |  |
| Married | 3609 (91.02%) | 2526 (91.03%) | 1083 (91.01%) |  |  |
| Residence, N(%) |  |  |  | 0.172 | 0.679 |
| Urban | 1694 (42.72%) | 1192 (42.95%) | 502 (42.18%) |  |  |
| Rural | 2271 (57.28%) | 1583 (57.05%) | 688 (57.82%) |  |  |
| Education level, N(%) |  |  |  | 2.299 | 0.513 |
| Below primary | 1743 (43.96%) | 1209 (43.57%) | 534 (44.87%) |  |  |
| Primary | 845 (21.31%) | 609 (21.95%) | 236 (19.83%) |  |  |
| Middle | 901 (22.72%) | 624 (22.49%) | 277 (23.28%) |  |  |
| High school or above | 476 (12.01%) | 333 (12.00%) | 143 (12.02%) |  |  |
| Smoking status, N(%) |  |  |  | 1.993 | 0.369 |
| Never | 2750 (69.36%) | 1912 (68.90%) | 838 (70.42%) |  |  |
| Former | 384 (9.68%) | 265 (9.55%) | 119 (10.00%) |  |  |
| Current | 831 (20.96%) | 598 (21.55%) | 233 (19.58%) |  |  |
| Drinking status, N(%) |  |  |  | 0.271 | 0.873 |
| Never | 2510 (63.30%) | 1750 (63.06%) | 760 (63.87%) |  |  |
| Former | 323 (8.15%) | 229 (8.25%) | 94 (7.90%) |  |  |
| Current | 1132 (28.55%) | 796 (28.68%) | 336 (28.24%) |  |  |
| Insurance, N(%) |  |  |  | 0.082 | 0.775 |
| No | 324 (8.17%) | 224 (8.07%) | 100 (8.40%) |  |  |
| Yes | 3641 (91.83%) | 2551 (91.93%) | 1090 (91.60%) |  |  |
| SBP, mmHg | 130.00 [117.50;145.00] | 130.50 [118.00;145.50] | 129.00 [116.50;144.50] | 0.977 | 0.329 |
| DBP, mmHg | 77.50 [70.00;86.00] | 78.00 [70.00;86.00] | 77.50 [70.00;86.00] | 0.737 | 0.461 |
| WC, cm | 93.00 [88.00;98.10] | 92.80 [88.00;98.00] | 93.00 [88.00;98.35] | -1.589 | 0.112 |
| ADL score | 0.00 [0.00;0.00] | 0.00 [0.00;0.00] | 0.00 [0.00;0.00] | 0.133 | 0.835 |
| CESD-10 | 7.00 [3.00;12.00] | 7.00 [3.00;12.00] | 6.00 [3.00;11.00] | 0.510 | 0.609 |
| Total cognition score | 13.00 [10.00;15.00] | 12.50 [10.00;15.00] | 13.00 [10.00;15.00] | -0.872 | 0.383 |
| Glucose, mg/dl | 104.58 [96.30;117.00] | 104.58 [96.48;116.82] | 104.40 [96.12;118.03] | -0.154 | 0.877 |
| Creatinine, mg/dl | 0.75 [0.64;0.88] | 0.75 [0.64;0.88] | 0.75 [0.66;0.87] | 0.365 | 0.715 |
| TC, mg/dl | 194.85 [170.88;221.14] | 195.62 [170.68;221.52] | 192.91 [170.88;219.98] | 0.553 | 0.580 |
| TG, mg/dl | 126.56 [89.39;185.85] | 126.56 [90.27;187.62] | 124.78 [88.50;179.66] | 1.447 | 0.148 |
| HDL-C, mg/dl | 45.23 [37.50;53.74] | 45.23 [37.50;53.74] | 45.04 [37.20;53.74] | 0.195 | 0.845 |
| LDL-C, mg/dl | 118.69 [96.26;142.27] | 118.69 [95.88;143.04] | 119.07 [97.81;141.40] | -0.160 | 0.873 |
| CRP, mg/l | 1.29 [0.70;2.53] | 1.29 [0.71;2.55] | 1.28 [0.67;2.47] | 0.958 | 0.338 |
| HbA1c,% | 5.20 [4.90;5.50] | 5.20 [4.90;5.50] | 5.20 [4.90;5.60] | -1.639 | 0.100 |
| UA, mg/dl | 4.42 [3.67;5.27] | 4.46 [3.68;5.30] | 4.36 [3.64;5.23] | 1.736 | 0.083 |
| Hypertension, N(%) |  |  |  | 0.743 | 0.389 |
| No | 2514 (63.40%) | 1747 (62.95%) | 767 (64.45%) |  |  |
| Yes | 1451 (36.60%) | 1028 (37.05%) | 423 (35.55%) |  |  |
| Mental illness, N(%) |  |  |  | 0.062 | 0.803 |
| No | 3916 (98.76%) | 2742 (98.81%) | 1174 (98.66%) |  |  |
| Yes | 49 (1.24%) | 33 (1.19%) | 16 (1.34%) |  |  |
| Arthritis, N(%) |  |  |  | 0.105 | 0.746 |
| No | 2549 (64.29%) | 1779 (64.11%) | 770 (64.71%) |  |  |
| Yes | 1416 (35.71%) | 996 (35.89%) | 420 (35.29%) |  |  |
| Dyslipidemia, N(%) |  |  |  | 0.016 | 0.900 |
| No | 3338 (84.19%) | 2338 (84.25%) | 1000 (84.03%) |  |  |
| Yes | 627 (15.81%) | 437 (15.75%) | 190 (15.97%) |  |  |
| Liver disease, N(%) |  |  |  | 3.663 | 0.056 |
| No | 3830 (96.60%) | 2670 (96.22%) | 1160 (97.48%) |  |  |
| Yes | 135 (3.40%) | 105 (3.78%) | 30 (2.52%) |  |  |
| Kidney disease, N(%) |  |  |  | 0.329 | 0.566 |
| No | 3727 (94.00%) | 2604 (93.84%) | 1123 (94.37%) |  |  |
| Yes | 238 (6.00%) | 171 (6.16%) | 67 (5.63%) |  |  |
| Memory disorder, N(%) |  |  |  | 0.015 | 0.902 |
| No | 3912 (98.66%) | 2737 (98.63%) | 1175 (98.74%) |  |  |
| Yes | 53 (1.34%) | 38 (1.37%) | 15 (1.26%) |  |  |
| Headache, N(%) |  |  |  | 0.005 | 0.946 |
| No | 3418 (86.20%) | 2391 (86.16%) | 1027 (86.30%) |  |  |
| Yes | 547 (13.80%) | 384 (13.84%) | 163 (13.70%) |  |  |
| Chest pain, N(%) |  |  |  | 0.054 | 0.817 |
| No | 3732 (94.12%) | 2614 (94.20%) | 1118 (93.95%) |  |  |
| Yes | 233 (5.88%) | 161 (5.80%) | 72 (6.05%) |  |  |
| Back pain, N(%) |  |  |  | 1.252 | 0.263 |
| No | 3599 (90.77%) | 2509 (90.41%) | 1090 (91.60%) |  |  |
| Yes | 366 (9.23%) | 266 (9.59%) | 100 (8.40%) |  |  |
| CMM, N(%) |  |  |  | 0.793 | 0.373 |
| No | 3810 (96.09%) | 2672 (96.29%) | 1138 (95.63%) |  |  |
| Yes | 155 (3.91%) | 103 (3.71%) | 52 (4.37%) |  |  |

SBP, systolic blood pressure; DBP, diastolic blood pressure; WC, waist circumference; ADL, activities of daily living; CESD-10, 10-item Center for Epidemiologic Studies Depression Scale; TC, total cholesterol; TG, triglycerides; HDL-C, high-density lipoprotein cholesterol; LDL-C, low-density lipoprotein cholesterol; CRP, C-reactive protein; HbA1c, hemoglobin A1c; UA, uric acid; CMM, cardiometabolic multimorbidity.

**Supplementary Table 2. Univariate Logistic regression results of predictors for CMM in the development dataset.**

| Variable | OR(95%CI) | Wald X^2^ | p value |
| --- | --- | --- | --- |
| Age | 1.047 (1.025-1.069) | 18.488 | <0.001 |
| Sex |  |  |  |
| Female | Ref(1.00) |  |  |
| Male | 0.995 (0.663-1.493) | 0.001 | 0.980 |
| Marital status |  |  |  |
| Other | Ref(1.00) |  |  |
| Married | 0.671 (0.369-1.218) | 1.721 | 0.190 |
| Residence |  |  |  |
| Urban | Ref(1.00) |  |  |
| Rural | 0.408 (0.271-0.615) | 18.355 | <0.001 |
| Education level |  |  |  |
| Below primary | Ref(1.00) |  |  |
| Primary | 0.760 (0.431-1.342) | 0.893 | 0.345 |
| Middle | 0.968 (0.575-1.629) | 0.015 | 0.901 |
| High school or above | 1.692 (0.983-2.912) | 3.601 | 0.058 |
| Smoking status |  |  |  |
| Never | Ref(1.00) |  |  |
| Former | 1.114 (0.565-2.196) | 0.098 | 0.755 |
| Current | 1.396 (0.887-2.195) | 2.083 | 0.149 |
| Drinking status |  |  |  |
| Never | Ref(1.00) |  |  |
| Former | 1.586 (0.878-2.867) | 2.336 | 0.126 |
| Current | 0.628 (0.379-1.040) | 3.263 | 0.071 |
| Insurance |  |  |  |
| No | Ref(1.00) |  |  |
| Yes | 0.914 (0.455-1.837) | 0.064 | 0.800 |
| SBP | 1.013 (1.005-1.022) | 9.015 | 0.003 |
| DBP | 1.007 (0.991-1.023) | 0.766 | 0.382 |
| WC | 0.998 (0.981-1.014) | 0.081 | 0.776 |
| ADL score | 1.454 (1.257-1.681) | 25.447 | <0.001 |
| CESD-10 | 1.074 (1.045-1.104) | 26.156 | <0.001 |
| Total cognition score | 0.978 (0.925-1.035) | 0.573 | 0.449 |
| Glucose | 1.012 (1.009-1.015) | 63.657 | <0.001 |
| Creatinine | 3.895 (1.958-7.749) | 15.013 | <0.001 |
| TC | 1.000 (0.995-1.005) | 0.017 | 0.896 |
| TG | 1.002 (1.0001-1.003) | 4.908 | 0.027 |
| HDL-C | 0.976 (0.960-0.993) | 8.043 | 0.005 |
| LDL-C | 0.997 (0.992-1.003) | 1.072 | 0.301 |
| CRP | 1.012 (0.995-1.030) | 1.817 | 0.178 |
| HbA1c | 1.747 (1.541-1.981) | 75.92 | <0.001 |
| UA | 1.109 (0.955-1.288) | 1.851 | 0.174 |
| Hypertension |  |  |  |
| No | Ref(1.00) |  |  |
| Yes | 6.325 (3.952-10.125) | 59.057 | <0.001 |
| Mental illness |  |  |  |
| No | Ref(1.00) |  |  |
| Yes | 1.687 (0.398-7.147) | 0.504 | 0.478 |
| Arthritis |  |  |  |
| No | Ref(1.00) |  |  |
| Yes | 2.108 (1.420-3.129) | 13.676 | <0.001 |
| Dyslipidemia |  |  |  |
| No | Ref(1.00) |  |  |
| Yes | 5.122 (3.427-7.655) | 63.499 | <0.001 |
| Liver disease |  |  |  |
| No | Ref(1.00) |  |  |
| Yes | 2.235 (1.057-4.73) | 4.426 | 0.035 |
| Kidney disease |  |  |  |
| No | Ref(1.00) |  |  |
| Yes | 2.986 (1.711-5.214) | 14.807 | <0.001 |
| Memory disorder |  |  |  |
| No | Ref(1.00) |  |  |
| Yes | 8.726 (4.017-18.953) | 29.965 | <0.001 |
| Headache |  |  |  |
| No | Ref(1.00) |  |  |
| Yes | 1.629 (0.996-2.664) | 3.779 | 0.052 |
| Chest pain |  |  |  |
| No | Ref(1.00) |  |  |
| Yes | 3.205 (1.833-5.604) | 16.689 | <0.001 |
| Back pain |  |  |  |
| No | Ref(1.00) |  |  |
| Yes | 1.381 (0.761-2.506) | 1.128 | 0.288 |

OR, odds ratio; CI, confidence interval; SBP, systolic blood pressure; DBP, diastolic blood pressure; WC, waist circumference; ADL, activities of daily living; CESD-10, 10-item Center for Epidemiologic Studies Depression Scale; TC, total cholesterol; TG, triglycerides; HDL-C, high-density lipoprotein cholesterol; LDL-C, low-density lipoprotein cholesterol; CRP, C-reactive protein; HbA1c, hemoglobin A1c; UA, uric acid; CMM, cardiometabolic multimorbidity.

**Supplementary Figure 1. The study’s design and flow chart.**

**Supplementary Figure 2. LASSO regression analysis for analytical screening of predictor variables.**

(A) The coefficient trajectory plot was generated along a sequence of log-transformed lambda values, where variables with non-zero coefficients at the optimal lambda were retained as predictors. **(B)**The optimal lambda parameter in the LASSO model was determined through tenfold cross-validation. A plot of the binomial deviance against log(lambda) was drawn, with a vertical line added at the optimal value selected by the one-standard-error (1-SE) rule.

***Supplementary Figure 3. ROC curves of the sensitivity analysis using dichotomized depressive symptoms for predicting CMM in overweight/obese middle-aged and older adults.*** *(A) ROC curve generated using the development dataset. (B) ROC curve generated using the validation dataset. (C) ROC curve generated using the HRS external validation dataset.*

***Supplementary Table 3. Harmonisation of outcome and predictor definitions between CHARLS and HRS.***

| ***Variable*** | ***CHARLS definition*** | ***HRS definition*** | ***Potential misclassification*** |
| --- | --- | --- | --- |
| *CMM* | *Two or more self-reported physician-diagnosed diabetes/high blood sugar, heart disease, and stroke.* | *Two or more self-reported physician-diagnosed diabetes, heart disease, and stroke/TIA.* | *Self-report may be affected by healthcare access and recall bias.* |
| *BMI* | *Measured BMI; BMI >=24 kg/m² for overweight/obesity.* | *Measured BMI; main analysis BMI >=24 kg/m²; sensitivity BMI >=25 kg/m².* | *Ethnic-specific BMI thresholds affect cross-national comparability.* |
| *Residence* | *Urban/rural household registration.* | *Urban/rural residence variable.* | *Urban-rural definitions differ between countries.* |
| *ADL score* | *Six-item ADL difficulty score.* | *Six-item ADL score harmonised to same direction.* | *Instrument harmonisation may not capture identical severity.* |
| *CESD* | *CESD-10 score, range 0-30.* | *Eight-item CESD score, range 0-8.* | *Different scale length may attenuate comparability.* |
| *HbA1c* | *Blood biomarker, continuous.* | *Blood biomarker, continuous.* | *Relatively objective measurement.* |
| *Hypertension* | *Self-reported physician diagnosis.* | *Self-reported physician diagnosis.* | *Self-report may under-detect undiagnosed disease.* |
| *Arthritis* | *Self-reported physician diagnosis.* | *Self-reported physician diagnosis.* | *Self-report may under-detect undiagnosed disease.* |
| *Dyslipidemia* | *Self-reported physician diagnosis.* | *Defined using TC and HDL-C clinical cut-offs.* | *Different definitions may introduce non-equivalent classification.* |
| *Memory disorder* | *Self-reported physician diagnosis.* | *Self-reported memory-related diagnosis.* | *Self-report may be influenced by diagnostic awareness.* |

*CMM, cardiometabolic multimorbidity; BMI, body mass index; HRS, Health and Retirement Study; CHARLS, China Health and Retirement Longitudinal Study; ADL, activities of daily living; CESD-10, 10-item Center for Epidemiologic Studies Depression Scale; CESD, Center for Epidemiologic Studies Depression Scale; HbA1c, hemoglobin A1c; TC, total cholesterol; HDL-C, high-density lipoprotein cholesterol; TIA, transient ischemic attack.*

***Supplementary Table 4. Agreement between self-reported and laboratory-defined dyslipidemia in the CHARLS cohort.***

|  | *N* | *Laboratory defined positive* | *Self reported positive* | *Kappa* | *Accuracy* | *Sensitivity* | *Specificity* | *PPV* | *NPV* |
| --- | --- | --- | --- | --- | --- | --- | --- | --- | --- |
| *Accuracy* | *3965* | *3039* | *627* | *0.034* | *0.341* | *0.173* | *0.891* | *0.839* | *0.247* |

*Cohen’s kappa coefficient; PPV, positive predictive value; NPV, negative predictive value. Laboratory-defined dyslipidemia was defined as total cholesterol (TC) >200 mg/dL and/or high-density lipoprotein cholesterol (HDL-C) <40 mg/dL in men or <50 mg/dL in women. Self-reported dyslipidemia was based on participants’ reports of a physician diagnosis of dyslipidemia. Sensitivity, specificity, PPV, and NPV were calculated using laboratory-defined dyslipidemia as the reference standard.*

***Supplementary Table 5. Variance inflation factors for predictors included in the final nomogram model.***

| ***Variable*** | ***VIF*** |
| --- | --- |
| *ADL score* | *1.130* |
| *CESD-10 score* | *1.154* |
| *HbA1c* | *1.017* |
| *Residence* | *1.025* |
| *Hypertension* | *1.078* |
| *Arthritis* | *1.073* |
| *Dyslipidemia* | *1.075* |
| *Memory disorder* | *1.025* |

*VIF, variance inflation factor; ADL, activities of daily living; CESD-10, 10-item Center for Epidemiologic Studies Depression Scale; HbA1c, hemoglobin A1c.*

***Supplementary Table 6. Discrimination of the full nomogram model and pared-down model.***

| ***Model*** | ***Development AUC*** | ***Validation AUC*** | ***External validation AUC*** |
| --- | --- | --- | --- |
| *Full nomogram model* | *0.871* | *0.817* | *0.733* |
| *Pared-down model* | *0.850* | *0.812* | *0.724* |

*AUC, area under the receiver operating characteristic curve.*

*Pared-down model refers to the simplified model excluding ADL score, CESD-10 score, and memory disorder.*

***Supplementary Table 7. Predictive values at practical thresholds in the development, validation, and external validation datasets.***

| ***Dataset*** | ***Threshold*** | ***Sensitivity*** | ***Specificity*** | ***PPV*** | ***NPV*** |
| --- | --- | --- | --- | --- | --- |
| *Development* | *0.033* | *0.874* | *0.760* | *0.123* | *0.994* |
| *Development* | *0.050* | *0.757* | *0.841* | *0.155* | *0.989* |
| *Development* | *0.100* | *0.524* | *0.929* | *0.222* | *0.981* |
| *Development* | *0.200* | *0.291* | *0.975* | *0.309* | *0.973* |
| *Validation* | *0.026* | *0.808* | *0.714* | *0.114* | *0.988* |
| *Validation* | *0.050* | *0.615* | *0.838* | *0.148* | *0.979* |
| *Validation* | *0.100* | *0.327* | *0.929* | *0.173* | *0.968* |
| *Validation* | *0.200* | *0.192* | *0.975* | *0.263* | *0.964* |
| *External validation* | *0.109* | *0.768* | *0.601* | *0.170* | *0.954* |
| *External validation* | *0.050* | *0.901* | *0.318* | *0.128* | *0.967* |
| *External validation* | *0.100* | *0.760* | *0.570* | *0.164* | *0.955* |
| *External validation* | *0.200* | *0.501* | *0.809* | *0.225* | *0.936* |

*PPV, positive predictive value; NPV, negative predictive value.*

***Supplementary Table 8. HRS external validation sensitivity analysis using BMI >=25 kg/m².***

| ***Dataset*** | ***N*** | ***CMM cases*** | ***AUC*** | ***Sensitivity*** | ***Specificity*** | ***PPV*** | ***NPV*** |
| --- | --- | --- | --- | --- | --- | --- | --- |
| *HRS BMI >=25 kg/m²* | *7,556* | *778* | *0.731* | *0.742* | *0.594* | *0.173* | *0.952* |

*HRS, Health and Retirement Study; BMI, body mass index; CMM, cardiometabolic multimorbidity; AUC, area under the receiver operating characteristic curve; PPV, positive predictive value; NPV, negative predictive value.*

***Supplementary Table 9. Model discrimination and calibration performance in the development, internal validation, and HRS external validation datasets***

| *Dataset* | *N* | *Events* | *Expected events* | *OE ratio* | *Brier score* | *Calibration intercept* | *Calibration slope* |
| --- | --- | --- | --- | --- | --- | --- | --- |
| *Development set* | *2775* | *103* | *103* | *1.000* | *0.0313* | *0.000* | *1.000* |
| *Validation set* | *1190* | *52* | *44.33* | *1.173* | *0.0412* | *0.203* | *0.713* |
| *HRS external validation* | *8180* | *816* | *538.9* | *1.514* | *0.0846* | *0.539* | *0.670* |
| *Intercept recalibrated model* | *8180* | *816* | *816* | *1.000* | *0.0842* | *0.000* | *0.670* |

*Abbreviations: O:E, observed-to-expected event ratio; HRS, Health and Retirement Study.*

***Supplementary Table 10. Variable-specific missing proportions before imputation among overweight/obese CHARLS participants (n=3,965).*** Laboratory biomarkers are complete (0%) by design, as participants with missing laboratory measurements were excluded during sample selection; the remaining continuous non-laboratory variables were handled by multiple imputation, whereas missing categorical disease-history and lifestyle responses were coded as absence of the condition.

| *Variable* | *Missing, n (%)* |
| --- | --- |
| *Age, years* | *11 (0.35)* |
| *Sex* | *0 (0.00)* |
| *Marital status* | *0 (0.00)* |
| *Residence* | *0 (0.00)* |
| *Education level* | *0 (0.00)* |
| *Smoking status* | *7 (0.18)* |
| *Drinking status* | *3 (0.08)* |
| *Insurance* | *98 (2.47)* |
| *SBP, mmHg* | *41 (1.03)* |
| *DBP, mmHg* | *40 (1.01)* |
| *WC, cm* | *6 (0.15)* |
| *ADL score* | *34 (0.86)* |
| *CESD-10* | *36 (0.91)* |
| *Total cognition score* | *663 (16.72)* |
| *Glucose, mg/dl* | *0 (0.00)* |
| *Creatinine, mg/dl* | *0 (0.00)* |
| *TC, mg/dl* | *0 (0.00)* |
| *TG, mg/dl* | *0 (0.00)* |
| *HDL-C, mg/dl* | *0 (0.00)* |
| *LDL-C, mg/dl* | *0 (0.00)* |
| *CRP, mg/l* | *0 (0.00)* |
| *HbA1c,%* | *0 (0.00)* |
| *UA, mg/dl* | *0 (0.00)* |
| *Hypertension* | *11 (0.28)* |
| *Mental illness* | *9 (0.27)* |
| *Arthritis* | *4 (0.11)* |
| *Dyslipidemia* | *62 (1.56)* |
| *Liver disease* | *13 (0.33)* |
| *Kidney disease* | *7 (0.18)* |
| *Memory disorder* | *4 (0.11)* |
| *Headache* | *6 (0.15)* |
| *Chest pain* | *6 (0.15)* |
| *Back pain* | *6 (0.15)* |
| *CMM* | *0 (0.00)* |

*SBP, systolic blood pressure; DBP, diastolic blood pressure; WC, waist circumference; ADL, activities of daily living; CESD-10, 10-item Center for Epidemiologic Studies Depression Scale; TC, total cholesterol; TG, triglycerides; HDL-C, high-density lipoprotein cholesterol; LDL-C, low-density lipoprotein cholesterol; CRP, C-reactive protein; HbA1c, hemoglobin A1c; UA, uric acid; CMM, cardiometabolic multimorbidity.*
